# Benchmarking $\mathbf {\Lambda }$NN three-body forces and first predictions for $A=3-5$ hypernuclei

**DOI:** 10.1140/epja/s10050-024-01474-5

**Published:** 2025-02-01

**Authors:** Hoai Le, Johann Haidenbauer, Hiroyuki Kamada, Michio Kohno, Ulf-G. Meißner, Kazuya Miyagawa, Andreas Nogga

**Affiliations:** 1 Institute for Advanced Simulation (IAS-4), Forschungszentrum Jülich, 52425 Jülich, Germany; 2 Department of Physics, Faculty of Engineering, Kyushu Institute of Technology, Kitakyushu, 804-8550 Japan; 3 Research Center for Nuclear Physics, Osaka University, Ibaraki, 567-0047 Japan; 4 Helmholtz-Institut für Strahlen- und Kernphysik and Bethe Center for Theoretical Physics, Universität Bonn, 53115 Bonn, Germany; 5 CASA, Forschungszentrum Jülich, 52425 Jülich, Germany

## Abstract

Explicit expressions for the leading chiral hyperon-nucleon-nucleon three-body forces have been derived by Petschauer et al (Phys Rev C93:014001, 2016). An important prerequisite for including these three-body forces in few- and many-body calculations is the accuracy and efficiency of their partial-wave decomposition. A careful benchmark of the $\Lambda$NN potential matrix elements, computed using two robust and efficient partial-wave decomposition methods, is presented. In addition, results of a first quantitative assessment for the contributions of ${\Lambda }$NN forces to the separation energies in $A=3-5$ hypernuclei are reported.

## Introduction

Few-nucleon systems have served as a crucial testing ground for our understanding of nucleon-nucleon (NN) and three-nucleon (3N) forces [1–10]. In the course of this, due to the complexity of the computational treatment of few-body systems and the goal of achieving accurate predictions using realistic nuclear forces, it has become standard to cross-compare results achieved with various methods and by independent research groups. Indeed, such benchmark studies have become an integral part of the advancement of microscopic few-nucleon calculations. For instance, in the past, benchmark results have been produced for nucleon-deuteron (N–d) scattering [11, 12], for N–d breakup [13], for the triton binding energy including $2\pi$ exchange three-nucleon forces [14], for the four-nucleon (4N) bound state [15] and for 4N scattering [16, 17].

Regarding strangeness nuclear physics, realistic calculations of ${\Lambda }$ hypernuclei including the full complexity of the ${\Lambda }$N-$\mathrm {\Sigma }$N interaction were first presented in [18, 19] for the hypertriton and in [20–22] $\hbox {for }^4_{\Lambda }$H $\hbox {and }^4_{\Lambda }$He. Both are momentum-space calculations based on the Faddeev- and Faddeev–Yakubovsky (FY) approaches, respectively. Very recently, the first calculations of the hypertriton separation energy including chiral ${\Lambda }$NN three-body forces (3BFs) [23] have been published [24, 25]. Actual benchmark studies for hypernuclei are however scarce. Over the years, a diverse range of calculations employing various methods [22, 26–34] have been carried out. However, the elementary NN and hyperon-nucleon (YN) potentials utilized as input in those calculations are very different, making a comprehensive comparison of the results not possible. On the other hand, an actual benchmark study for few-body hypernuclei presented in Ref. [35] relied on rather simple representations of the NN and YN interactions. Only lately, first elaborate benchmark results $\hbox {for }^4_{\Lambda }$H [33] were reported, by comparing calculations based on the FY equations and the Jacobi no-core shell model (Jacobi-NCSM), for state-of-the-art NN and YN two-body interactions, namely the so-called SMS NN potentials derived within chiral effective field theory (EFT) [36] and YN interactions established likewise in chiral EFT [37, 38].

With the present work we want to add a further benchmark for ${\Lambda }$ hypernuclei. Specifically, we provide a detailed comparison of the calculations by Kamada, Kohno, and Miyagawa (KKM) [24, 25] and the Jülich–Bonn Group (JBG) [39, 40] for the hypertriton including chiral 3BFs. The former calculation is performed within the Faddeev approach while the latter utilizes the Jacobi-NCSM formalism. The motivation for our study originates from discrepancies in the contribution of the $2\pi$ exchange ${\Lambda }$NN force to the hypertriton separation energy observed between the KKM results [24] and the preparatory calculations of JBG. In the course of clarifying them, see errata to Refs. [24, 25, 41], it became clear that it would be rather useful to provide an in-depth comparison of the results by the two groups, which does not only shed light on the accuracy of the two methods but also allows for an examination of the underlying technical and numerical aspects of such complex calculations. Clearly, such a detailed comparison is not only indispensable for corroborating the outcome of the present three-body calculations, but it provides also a useful guideline for future calculations employing different few-body methods.

The paper is organized as follow. In the following section, we briefly describe the two approaches for the partial-wave decomposition of the ${\Lambda }$NN (and $\mathrm {\Sigma }$NN) potentials employed by KKM and JBG. A detailed comparison of the ${\Lambda }$NN potential matrix elements in different partial-wave states are presented in Sect. 3. In Sect. 4 we discuss possible contributions of the chiral ${\Lambda }$NN interaction to the separation energies in the $A=3-5$ hypernuclei and we close with some concluding remarks.

## Partial-wave decomposition of the chiral $\textrm{YNN}$ forces

The generic contact, one- and two-meson exchange diagrams for the process $B_1 B_2 B_3 \rightarrow B_4B_5 B_6$, appearing at next-to-next-to-leading order (N$^2$LO) in the chiral expansion [23], are shown in panels (a), (b) and (c) in Fig. 1, respectively. The fully antisymmetrized contact $\textrm{YNN}$ potential, obtained from the diagram (a) in Fig. 1 and all the permutations of the incoming $B_1 B_2 B_3$ and outgoing $B_4 B_5 B_6$ baryon states, is given by [23, 42]

1 $$\begin{aligned} \begin{aligned} V_\textrm{ct}&= -\Bigg [ N^1_{\begin{array}{c} 456\\ 123 \end{array}} + N^2_{\begin{array}{c} 456 \\ 123 \end{array}}\vec {\sigma }_A . \vec {\sigma }_B + N^3_{\begin{array}{c} 456 \\ 123 \end{array}}\vec {\sigma }_A . \vec {\sigma }_C\\&\qquad + N^4_{\begin{array}{c} 456 \\ 123 \end{array}}\vec {\sigma }_B . \vec {\sigma }_C + N^5_{\begin{array}{c} 456 \\ 123 \end{array}} i \vec {\sigma }_A . (\vec {\sigma }_B \times \vec {\sigma }_C) \Bigg ] , \end{aligned}\nonumber \\ \end{aligned}$$

where $$N^{i}_{\begin{array}{c} 456 \\ 123 \end{array}}$$ are appropriately antisymmetrized combinations of the 18 LECs defined in Eq. (18) of Ref. [23]. The one-meson exchange potential corresponding to the master diagram (b) in Fig. 1 reads,

2 $$\begin{aligned} \begin{aligned} V_\textrm{1me}&= \frac{1}{2f^2_{0}} \frac{\vec {\sigma }_A .\vec {q}_{li}}{{\vec {q}_{li}}^{\, 2} + m^2_{\phi }}\Big [ N_1 \vec {\sigma }_C . \vec {q}_{li} + N_2 i (\vec {\sigma }_B \times \vec {\sigma }_C) \vec {q}_{li} \Big ] , \end{aligned} \end{aligned}$$

with $\vec {q}_{li} =\vec {p}_l - \vec {p}_i$ the transferred momentum. Explicit expressions for the constants $N_1, N_2$ are given by Eq. (30) in Ref. [23]. Based on the general expression in Eq. (2), the antisymmetrized one-meson exchange $\mathrm {B_1 B_2 B_3} \rightarrow \mathrm {B_4 B_5 B_6 }$ potential can be obtained by summing up for each exchange meson $\phi$ the 36 permutations of the initial and final baryons. Finally, the two-meson exchange diagram (c) yields

3 $$\begin{aligned} \begin{aligned} V_\textrm{2me} =&-\frac{1}{4f_0^4} \frac{\vec {\sigma }_A. \vec {q}_{li} \, \vec {\sigma }_C . \vec {q}_{nk}}{ (\vec {q}^{\, 2}_{li} + m^2_{\phi _1}) (\vec {q}^{\, 2}_{nk} +m^2_{\phi _2})}\\&\times [ N^{\prime }_1 + N^{\prime }_2 \vec {q}_{li} . \vec {q}_{nk} + N^{\prime }_3 i (\vec {q}_{li} \times \vec {q}_{nk}) .\vec {\sigma }_B] . \end{aligned} \nonumber \\ \end{aligned}$$

The constants $N^{\prime }_{1,2,3}$ are defined in Eq. (34) in Ref. [23]. Similarly, summing up Eq. (3) for all the 18 permutations^1^ of the initial and final baryon states and all possible exchanged mesons, one obtains the general antisymmetrized two-meson exchange YNN potential. Note that, in the calculations by JBG, all the coefficients $$N^{i}_{\begin{array}{c} 456 \\ 123 \end{array}}$$, $N_{i}$ and $N^{\prime }_{i}$ in Eqs. (1–3) have been evaluated as functions of the involved LECs using *Mathematica*.

**Fig. 2:**
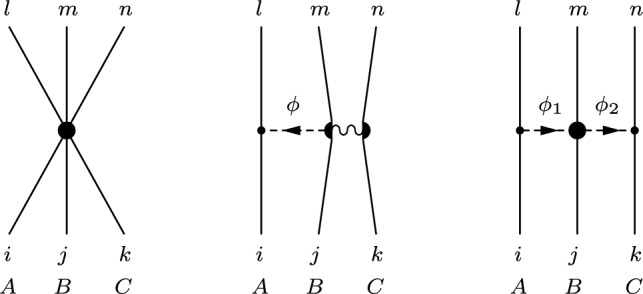
Generic $\textrm{YNN} \rightarrow \textrm{YNN}$ diagrams: **a** contact term, **b** one-meson exchange, **c** two-meson exchange. The wiggly line symbolizes the four-baryon contact vertex, to illustrate the baryon bilinears

For the case of $\Lambda \textrm{NN} \rightarrow \Lambda \textrm{NN}$ 3BFs that involve only $\pi$-meson exchanges, the expressions for the $V^{\Lambda \textrm{NN}}$ potentials in Eqs. (1–3) can be significantly simplified [23],

4 $$\begin{aligned} V^{\Lambda \textrm{NN}}_{ct}= &+ C'_1\ (\mathbbm {1} - \mathbf {\sigma }_2\cdot \mathbf {\sigma }_3 ) ( 3 + \mathbf {\tau }_2\cdot \mathbf {\tau }_3 ) \nonumber \\&+ C'_2\ \mathbf {\sigma }_1\cdot (\mathbf {\sigma }_2+\mathbf {\sigma }_3)\,(\mathbbm {1} - \mathbf {\tau }_2\cdot \mathbf {\tau }_3) \nonumber \\&+ C'_3\ (3 + \mathbf {\sigma }_2\cdot \mathbf {\sigma }_3 ) ( \mathbbm {1} - \mathbf {\tau }_2\cdot \mathbf {\tau }_3 ) \,, \end{aligned}$$

5 $$\begin{aligned} V^{\Lambda \textrm{NN}}_{1\pi }=&  -\frac{g_A}{2f_0^2} \,\bigg ( \frac{\mathbf {\sigma }_2\cdot \textbf{q}_{52}}{\textbf{q}_{52}^{\,2}+m_\pi ^2} \mathbf {\tau }_2\cdot \mathbf {\tau }_3 \Big [ (D'_1\mathbf {\sigma }_1+D'_2\mathbf {\sigma }_3)\cdot \textbf{q}_{52} \Big ] \nonumber \\&+\frac{\mathbf {\sigma }_3\cdot \textbf{q}_{63}}{\textbf{q}_{63}^{\,2}+m_\pi ^2} \mathbf {\tau }_2\cdot \mathbf {\tau }_3 \Big [ (D'_1\mathbf {\sigma }_1+D'_2\mathbf {\sigma }_2)\cdot \textbf{q}_{63} \Big ] \nonumber \\&+P^{(\sigma )}_{23}P^{(\tau )}_{23} P^{(\sigma )}_{13}\frac{\mathbf {\sigma }_2\cdot \textbf{q}_{62}}{\textbf{q}_{62}^{\,2}+m_\pi ^2} \mathbf {\tau }_2\cdot \mathbf {\tau }_3 \nonumber \\&\times \Bigg [ -\frac{D'_1+D'_2}{2} (\mathbf {\sigma }_1+\mathbf {\sigma }_3)\cdot \textbf{q}_{62} \nonumber \\&\quad \qquad + \frac{D'_1-D'_2}{2}\,\mathrm i\,(\mathbf {\sigma }_3\times \mathbf {\sigma }_1)\cdot \textbf{q}_{62} \Bigg ] \nonumber \\&+P^{(\sigma )}_{23}P^{(\tau )}_{23} P^{(\sigma )}_{12}\frac{\mathbf {\sigma }_3\cdot \textbf{q}_{53}}{\textbf{q}_{53}^{\,2}+m_\pi ^2} \mathbf {\tau }_2\cdot \mathbf {\tau }_3 \nonumber \\&\times \Bigg [ -\frac{D'_1+D'_2}{2} (\mathbf {\sigma }_1+\mathbf {\sigma }_2)\cdot \textbf{q}_{53} \nonumber \\&\quad \qquad - \frac{D'_1-D'_2}{2}\,\mathrm i\,(\mathbf {\sigma }_1\times \mathbf {\sigma }_2)\cdot \textbf{q}_{53} \Bigg ] \bigg )\,, \end{aligned}$$

and,

6 $$\begin{aligned} V^{\Lambda \textrm{NN}}_{2\pi }&= \frac{g_A^2}{3f_0^4} \frac{\mathbf {\sigma }_3\cdot \textbf{q}_{63}\ \mathbf {\sigma }_2\cdot \textbf{q}_{52}}{(\textbf{q}_{63}^{\,2}+m_{\pi }^2)(\textbf{q}_{52}^{\,2}+m_{\pi }^2)} \mathbf {\tau }_2\cdot \mathbf {\tau }_3 \nonumber \\&\quad \times \Big ( -(3 b_0 + b_D) m_\pi ^2 + (2 b_2 + 3 b_4) \,\textbf{q}_{63}\cdot \textbf{q}_{52}\Big ) \nonumber \\&\quad - P^{(\sigma )}_{23}P^{(\tau )}_{23} \frac{g_A^2}{3f_0^4} \frac{\mathbf {\sigma }_3\cdot \textbf{q}_{53}\ \mathbf {\sigma }_2\cdot \textbf{q}_{62}}{(\textbf{q}_{53}^{\,2}+m_{\pi }^2)(\textbf{q}_{62}^{\,2}+m_{\pi }^2)} \mathbf {\tau }_2\cdot \mathbf {\tau }_3 \nonumber \\&\quad \times \Big ( -(3 b_0 + b_D) m_\pi ^2 + (2 b_2 + 3 b_4) \,\textbf{q}_{53}\cdot \textbf{q}_{62}\Big ),\nonumber \\ \end{aligned}$$

with appropriate exchange operators in spin and isospin space, $P^{(\sigma )}_{ij}=\frac{1}{2}(\mathbbm {1}+\mathbf {\sigma }_i\cdot \mathbf {\sigma }_j)$, $P^{(\tau )}_{ij}=\frac{1}{2}(\mathbbm {1}+\mathbf {\tau }_i\cdot \mathbf {\tau }_j)$. Here $f_0$ and $g_A$ are the Goldstone boson decay constant and the nucleon axial-vector coupling constant, where we use $f_0=93$ MeV and $g_A=1.26$. The quantities $C_i', D_i', b_i$ are low-energy constants (LECs), the latter can in principle be fixed from the octet baryon masses and three-flavor meson-baryon scattering [43]. Note that, when the potentials in Eqs. (1–6) are applied to basis wave functions $\text {YNN}$ ($\Lambda \text {NN}$) for which the two-nucleon states are antisymmetric, a scaling factor of $\frac{1}{2}$ [24, 25, 41] is required.

In order to include the above $\textrm{YNN}$ (${\Lambda \textrm{NN}}$) interactions in few- and many-body hypernuclear calculations, efficient and accurate methods for the partial-wave decomposition of these potentials are of importance. Therefore, in this study, we want to benchmark the chiral potential matrix elements $V^{{\Lambda NN}}$ evaluated using two different partial-wave decomposition methods. In the first approach, referred to as lPWD, the locality of the chiral ${\Lambda \textrm{NN}}$ potentials in Eqs. (4–6) is explicitly exploited so that the eight-fold integration over the angles of the incoming and outgoing momenta can be reduced to a two-fold integration, which in turn can significantly speed up the generation of the 3BF matrix elements. This method has initially been applied to the local chiral 3NFs up to $\mathrm {N^3LO}$ by Hebeler et al. [44], and recently extended by KKM [41] to compute the partial-wave decomposition matrix elements of the chiral $\Lambda$NN 3BF at $\mathrm {N^2LO}$ based on Eqs. (4–6). In the method of KKM, the $\Lambda$NN interactions are rewritten in the tensor product form by separating the spin and angular-momentum parts. A convenient expression in a form similar to the Wigner–Eckart theorem is derived for the matrix element of the angle-dependent term. For more details, one can refer to [41]. In the second approach, utilized by JBG and referred to as aPWD, the technique introduced by Skibinski et al. in Ref. [45] is employed to automatically perform the partial-wave decomposition of both $\Lambda$NN and $\mathrm {\Sigma NN}$ potentials using the general expressions in Eqs. (1–3).

In general, the three-body $\textrm{YNN}$ partial-wave states $|p_{12} q_3\alpha _{\textrm{YNN}}\rangle$ with the total angular momentum *J* and total isospin *T* in *jj*-coupling can be constructed as follows

7 $$\begin{aligned} \begin{aligned} |p_{12} q_{3}\alpha _{\textrm{YNN}} \rangle&=| p_{12} q_3 (l_{12} s_{12})j_{12} \left( l_3 \frac{1}{2}\right) \\&\quad I_3 (j_{12} I_3) J M_J, (t_{12} t_{\textrm{Y}}) T M_T \rangle , \end{aligned} \end{aligned}$$

where $p_{12}$ and $q_3$ are the relative Jacobi momenta between two nucleons and between the center-of-mass of two nucleons and the hyperon, respectively, and $\alpha _{\textrm{YNN}}$ denotes a set of discrete quantum numbers characterizing the state. In the first step of the aPWD, the 3BF $\textrm{YNN}$ matrix elements are calculated in the partial-wave state in *LS*-coupling, $|p_{12} q_3 \beta _{\textrm{YNN}}\rangle$,

8 $$\begin{aligned} \begin{aligned} |p_{12} q_{3}\beta _{\textrm{YNN}} \rangle&=| p_{12} q_3 (l_{12} l_3) L \left( s_{12} \frac{1}{2}\right) S\\&\qquad (L,S) J M_{J}, (t_{12} t_{\textrm{Y}}) T M_{T} \rangle . \end{aligned} \end{aligned}$$

The *LS*-coupling representation $|p_{12} q_{3}\beta _{\textrm{YNN}} \rangle$ is related to the basis $|p_{12} q_{3}\alpha _{\textrm{YNN}}\rangle$ in Eq. (7) simply via a 9*j* symbol and Clebsch-Gordan coefficients [46]. In the basis $|p_{12} q_{3}\beta _{\textrm{YNN}} \rangle$, the 3BF $\textrm{YNN}$ matrix elements can be expressed as

9 $$\begin{aligned} \begin{aligned}&\langle p_{12}^{\prime } q_{3}^{\prime } \beta ^{\prime }_{\textrm{YNN}} | V_{\textrm{YNN}} | p_{12} q_{3} \beta _{\textrm{YNN}} \rangle \\&\quad = \int d\hat{p}_{12}^{\prime } \int d\hat{q}_3^{\prime } \int d\hat{p}_{12} \int d\hat{q}_3\\  &\qquad \sum _{m_{L^{\prime }}} C(L^{\prime } S^{\prime } J^{\prime }; m_{L^{\prime }}, M_{J^{\prime }} -m_{L^{\prime }}, M_{J^{\prime }}) \mathcal {Y}^{* L^{\prime }, m_{L^{\prime }}}_{l^{\prime }_{12} l^{\prime }_{3}}(\hat{p}_{12}^{\prime } \hat{q}_3^{\prime })\\  &\qquad \sum _{m_{L}} C(L S J; m_{L}, M_{J} -m_{L}, M_{J}) \mathcal {Y}^{L, m_{L}}_{l_{12} l_{3}}(\hat{p}_{12} \hat{q}_3)\\&\qquad \times \Bigg \langle p^{\prime }_{12} q^{\prime }_{3}\,\left( s^{\prime }_{12} \frac{1}{2}\right) S^{\prime } M_{J} - m_{L^{\prime }} (t^{\prime }_{12}, t_{Y^{\prime }}) T^{\prime } M_{T} | V^{\textrm{YNN}} | \\  &\qquad p_{12} q_{3} \,\left( s_{12} \frac{1}{2}\right) S M_{J} - m_{L} (t_{12}, t_{Y}) T M_{T} \Bigg \rangle , \end{aligned} \end{aligned}$$

where

10 $$\begin{aligned} \begin{aligned} \mathcal {Y}_{l_{12}l_3}^{L m_{L}}(\hat{p}_{12} \hat{q}_3) =&\!\!\!\sum _{m_{l_{12}}=-l_{12}}^{l_{12}}\!\!\!\! C(l_{12}, l_3, L; m_{l_{12}}, m_L -m_{l_{12}},m_L )\\  &\quad \times Y_{l_{12},m_{l_{12}}}(\hat{p}_{12}) Y_{l_3, m_L - m_{l_{12}} }(\hat{q}_{3}). \end{aligned} \end{aligned}$$

the matrix elements in the spin- and isospin-spaces in Eq. (9), $\Big \langle p^{\prime }_{12} q^{\prime }_{3}\,(s^{\prime }_{12} \frac{1}{2})S^{\prime } M_{S^{\prime }} (t^{\prime }_{12}, t_{Y^{\prime }}) T^{\prime } M_{T} | V^{\textrm{YNN}} |$ $p_{12} q_{3} \,(s_{12} \frac{1}{2})S M_{S} (t_{12}, t_{Y}) T M_{T} \rangle$, depend on the momenta, spin- and isospin-quantum numbers of the incoming and outgoing states. They can be computed in analytic form as a function of the momenta $p_{12}, q_{3}$ and $p^{\prime }_{12}, q^{\prime }_3$ for all combinations of spin and isospin- quantum numbers utilizing a software for symbolic calculations such as *Maple* (in our case) or *Mathematica* [45]. This symbolic software also allows an automatic generation of a FORTRAN code for these matrix elements, so that the multifold integration over the angular part in Eq. (9) can efficiently be calculated numerically using a FORTRAN program. Furthermore, given that the 3BFs $V^{\textrm{YNN}}$ is rotationally invariant, the matrix elements in Eq. (9) vanish unless $J= J^{\prime }$ and $M_{J} = M_{J^{\prime }}$, and in addition, they do not depend on the magnetic quantum number $M_{J}$, hence

11 $$\begin{aligned} \begin{aligned}&\langle p_{12}^{\prime } q_{3}^{\prime } \beta ^{\prime }_{\textrm{YNN}} | V_{\textrm{YNN}} | p_{12} q_{3} \beta _{\textrm{YNN}} \rangle \\&\quad = \int d\hat{p}_{12}^{\prime } \int d\hat{q}_3^{\prime } \int d\hat{p}_{12} \int d\hat{q}_3\, \frac{1}{2J+1} \sum _{m_{J}=-J}^{J}\\  &\quad \sum _{m_{L^{\prime }}} C(L^{\prime } S^{\prime } J; m_{L^{\prime }}, M_{J} -m_{L^{\prime }}, M_{J}) \mathcal {Y}^{* L^{\prime }, m_{L^{\prime }}}_{l^{\prime }_{12} l^{\prime }_{3}}(\hat{p}_{12}^{\prime } \hat{q}_3^{\prime })\\  &\quad \sum _{m_{L}} C(L S J; m_{L}, M_{J} -m_{L}, M_{J}) \mathcal {Y}^{ L, m_{L}}_{l_{12} l_{3}}(\hat{p}_{12} \hat{q}_3)\\  &\quad \times \Bigg \langle p^{\prime }_{12} q^{\prime }_{3}\left( s^{\prime }_{12} \frac{1}{2}\right) S^{\prime } M_{J} - m_{L^{\prime }} (t^{\prime }_{12}, t_{Y^{\prime }}) T^{\prime } M_{T} | V^{\textrm{YNN}} | \\  &\quad p_{12} q_{3} \left( s_{12} \frac{1}{2}\right) S M_{J} - m_{L} (t_{12}, t_{Y}) T M_{T} \Bigg \rangle . \end{aligned}\nonumber \\ \end{aligned}$$

Since the integrand in Eq. (11) is a scalar, one can freely chose the directions of the momenta, say $p^{\prime }_{12}$ and the azimuthal angle of $\textbf{q}_3$ is $q^{\prime }_{q3}$ such that $\vec {p}_{12} = (0,0,p_{12})$ and $\phi _{q_3} =0$. As a consequence, the eight-fold integration in Eq. (11) can be effectively reduced to a five-fold integration over the polar angle of $\textbf{q}_3$ and the solid angles $\hat{p}_{12}$ and $\hat{q}_3$ [45],

$$ \int d\hat{p}_{12}^{\prime } \int d\hat{q}_{3}^{\prime } \int d\hat{p}_{12} \int d\hat{q}_3 \rightarrow \int d \theta _{q_3}\int d\hat{p}_{12} \int d\hat{q}_3~, $$

which, in turn, can lead to a significant speed-up of the generation of the 3BF matrix-elements. Once the 3BF matrix elements in the *LS*-representation are known, the recoupling to the *jj*-basis, $\langle p_{12}^{\prime } q_{3}^{\prime } \alpha ^{\prime }_{\textrm{YNN}} | V_{\textrm{YNN}} | p_{12} q_{3} \alpha _{\textrm{YNN}} \rangle$, can easily be done [46]. In addition, since we assume that the 3BF YNN is charge independent, it is sufficient to compute the matrix elements in Eq. (11) for a specific value of $m_T$, say $m_{T} =0$.

## Benchmarking ${\Lambda }$NN matrix elements

We are now in a position to benchmark the 3BF matrix elements computed using the two different partial-wave decomposition approaches described in the previous section. Since the lPWD method has only been implemented for the $\Lambda$NN potential, we will focus on comparing the $\Lambda$NN potential matrix elements and turn off the $\mathrm {\Sigma }$ components in the aPWD approach also for the binding energy calculations discussed later. In Table 1, we list the quantum numbers of the $\alpha _{\Lambda \textrm{NN}}$ states with positive parity and the total angular momentum and isospin of $(J^{\pi }, T) = (1/2^+,0)$ and $(3/2^+,0)$ which have been selected for benchmarking. The $2\pi$-exchange $\Lambda$NN matrix elements, $\langle p^{\prime }_{12} q^{\prime }_{3} \alpha _{\Lambda \textrm{NN}} ' | V_{2\pi } | p_{12} q_3 \alpha _{{\Lambda \textrm{NN}}} \rangle$, computed at fixed Jacobi momenta

$p^{\prime }_{12} = p_{12} =q_{3} = 0.205507$ fm^–1^ and $q^{\prime }_{3} = 0.306967$ fm^–1^ are presented in Table 2. The sub-leading meson-baryon LECs [23], appearing in $V^{\Lambda \textrm{NN}}_\mathrm {2\pi }$, have been set to $3b_0 + b_D = 0$ and $2b_2 + 3b_4 = -3.0\times 10^{-3}$ MeV^–1^. One can clearly observe an almost perfect agreement between the aPWD and lPWD $2\pi$-exchange ${\Lambda \textrm{NN}}$ matrix elements.

**Table 1:** Quantum numbers of the first three ${\Lambda }$NN partial-wave states for the two selected $J^\pi$ and *T*

$(\textrm{J}^{\pi },T)$	$\alpha _{{\Lambda \textrm{NN}}}$	$l_{\textrm{12}}$	$s_{\textrm{12}}$	$J_{\textrm{12}}$	$t_{\textrm{12}}$	$l_{{\Lambda }}$	$2I_{{\Lambda }}$
$(1/2^+,0)$	1	0	1	1	0	0	1
	2	2	1	1	0	0	1
	3	1	0	1	0	1	1
$(3/2^+,0)$	1	0	1	1	0	0	1
	2	2	1	1	0	0	1
	3	1	0	1	0	1	1

**Table 2:** 2$\pi$-exchange ${\Lambda }$NN matrix elements $\langle p^{\prime }_{12} q^{\prime }_{3} \alpha ^{\prime }_{\Lambda \textrm{NN}} | V_{\mathrm {2\pi }} | p_{12} q_3 \alpha _{\Lambda \textrm{NN}} \rangle$ in fm^5^, computed with the automatic partial-wave decomposition (aPWD) and the approach that exploits the locality of the chiral YNN interaction (lPWD). The incoming and outgoing momenta are fixed to $p^{\prime }_{12} = p_{12} = q_3 = 0.205507$ fm^–1^ and $q^{\prime }_3 =0.306967$ fm^–1^. The sub-leading meson-baryon LECs [23] are set to $3b_0 + b_D = 0$ and $2b_2 + 3b_4 = -3.0\times 10^{-3}$ MeV^–1^

$\alpha ^{\prime }_{\Lambda \textrm{NN}}$	$\alpha _{\Lambda \textrm{NN}}$	J = 1/$2^{+}$, T = 0			J = 3/$2^{+}$, T = 0		
		aPWD	lPWD	Diff [%]	aPWD	lPWD	diff [%]
1	1	0.211808E$-$03	0.211795E$-$03	0.01	0.211818E$-$03	0.211795E$-$03	0.01
2	1	0.488366E$-$03	0.488674E$-$03	0.06	0.488367E$-$03	0.488674E$-$03	0.06
3	1	0.200297E$-$03	0.200317E$-$03	0.01	$-$0.100145E$-$03	$-$0.100158E$-$03	0.01
1	2	0.488614E$-$03	0.488674E$-$03	0.01	0.488511E$-$03	0.488674E$-$03	0.03
2	2	$-$0.781242E$-$04	$-$0.781013E$-$04	0.03	$-$0.781352E$-$04	$-$0.781013E$-$04	0.04
3	2	0.504514E$-$04	0.504487E$-$04	0.01	$-$0.252244E$-$04	$-$0.252244E$-$04	0.00
1	3	0.112725E$-$03	0.112723E$-$03	0.002	$-$0.563600E$-$04	$-$0.563617E$-$04	0.03
2	3	0.341903E$-$04	0.341810E$-$04	0.03	$-$0.170948E$-$04	$-$0.170905E$-$04	0.02
3	3	0.779062E$-$04	0.779012E$-$04	0.01	0.779025E$-$04	0.779012E$-$04	0.02

**Table 3:** Contact and 1$\pi$-exchange ${\Lambda }$NN matrix elements in fm^5^, computed with the automatic partial-wave decomposition (aPWD) and the approach that exploits the locality of the chiral YNN interaction (lPWD). The incoming and outgoing momenta are fixed to $p^{\prime }_{12} = p_{12} = q_3 = 0.205507$ fm^–1^ and $q^{\prime }_3 =0.306967$ fm^–1^. LECs are set to $D^{\prime }_1 =0$, $D^{\prime }_2 = \frac{2 C }{9 f_0^2 \Delta } = 0.6268$ $\hbox {fm }^3$ with $C=3/4\, g_A$ and $\Delta =300$ MeV, and $C^{\prime }_2=0$, $C^{\prime }_1 = C^{\prime }_3 = \frac{1}{72 f_0^4 \Delta } = 0.1852$ $\hbox {fm }^5$

J=1/$$2^{+}$$, T = 0		$$V_{1\pi }$$			$$V_{ct} $$		
$\alpha ^{\prime }_{\Lambda \textrm{NN}}$	$\alpha _{\Lambda \textrm{NN}}$	aPWD	lPWD	Diff [%]	aPWD	lPWD	Diff [%]
1	1	0.166474E$-$02	0.167123E$-$02	0.4	0.379766E$-$02	0.380185E$-$02	0.1
2	1	0.156132E$-$02	0.156852E$-$02	0.4	0.0	0.0	
3	1	$-$0.27E$-$12	0.0		0.0	0.0	
1	2	0.156197E$-$02	0.156852E$-$02	0.4	0.0	0.0	
2	2	0.479602E$-$02	0.481549E$-$02	0.4	$-$0.25E$-$08	$-$0.91E$-$10	
3	2	0.48E$-$13	0.0		$-$0.18E$-$13	0.0	
1	3	0.32E$-$13	0.0		0.0	0.0	
2	3	0.86E$-$13	0.0		0.0	0.0	
3	3	0.503546E$-$04	0.505587E$-$04	0.4	$-$0.0	0.60E$-$19	

**Fig. 3:**
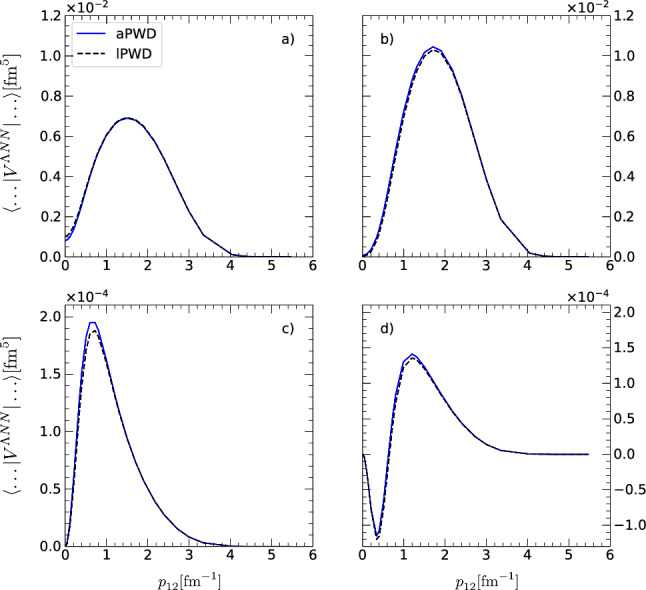
$2\pi$ (right panels) and $1\pi$ (left panels) ${\Lambda \textrm{NN}}$ matrix elements $\langle p^{\prime }_{12}\approx 0.206\,\textrm{fm}^{-1}, q^{\prime }_{3}\approx 0.206\,\textrm{fm}^{-1}, \alpha ^{\prime }_{\Lambda \textrm{NN}} | V^{{\Lambda \textrm{NN}}} | p_{12}, q_3 \approx 0.206\,\textrm{fm}^{-1}, \alpha _{{\Lambda \textrm{NN}}} \rangle$, computed using aPWD (solid lines) and lPWD (dashed line), as a function of $p_{12}$ in the $(J^{\pi },T)=(1/2^+,0)$ partial-wave state and for $(\alpha ^{\prime }_{{\Lambda \textrm{NN}}},\alpha _{{\Lambda \textrm{NN}}})$: **a**, **b** (1,1), **c**, **d** (2,2). All matrix have been regularized with a cutoff of $\Lambda =550$ MeV

**Fig. 4:**
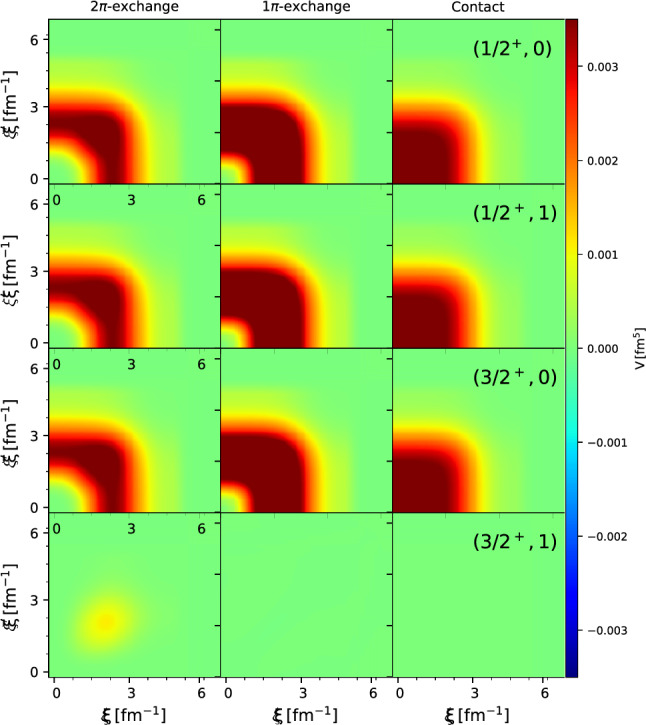
Matrix elements of 2$\pi$-, 1$\pi$- and contact- ${\Lambda \textrm{NN}}$ potentials $\langle p^{\prime }_{12} q^{\prime }_{3} \alpha ^{\prime }_{\Lambda \textrm{NN}} | V_{\Lambda \textrm{NN}} | p_{12} q_3 \alpha _{\Lambda \textrm{NN}} \rangle$ as a function of the hypermomentum $\xi ^2 = p^2_{12} + \frac{3}{4}q_3^2$ at hyperangles $\tan \theta = \frac{2}{\sqrt{3}}\,p_{12}/q_3 = \frac{\pi }{4}$ and $\tan \theta ' = \frac{\pi }{4}$ in different partial-wave states with $(J^{\pi }, T)= (1/2^+,0), (1/2^+,1), (3/2^+,0), (3/2^+,1)$. All matrix elements are in fm^5^ and have been regularized with a cutoff of $\Lambda =550$ MeV. The matrix elements in the partial wave state $(J^{\pi }, T)= (3/2^+,1)$ have been multiplied by a factor of 10 in order to make them visible

Table 3 lists the $1\pi$-exchange and contact ${\Lambda \textrm{NN}}$ matrix elements in the partial-wave state with $(J^{\pi },T) = (1/2^+, 0)$. The LECs in Eqs. (4, 5) are set to $D^{\prime }_1 =0$, $D^{\prime }_2 = \frac{2 C }{9 f_0^2 \Delta } = 0.6268$ fm^3^ with $C=\frac{3}{4}\, g_A=0.9525$,

$\Delta =300\,$MeV and $C^{\prime }_2=0$, $C^{\prime }_1 = C^{\prime }_3 = \frac{1}{72 f_0^4 \Delta } = 0.1852$ fm^5^ based on the so-called decuplet saturation scheme, see next section for details. We do not show here the results in the $(J^{\pi },T) = (3/2^+, 0)$ state but stress that similar agreement of better than $0.5\%$ is also observed for the $1\pi$-exchange and contact potential matrix elements in this partial-wave state. Figure 2 shows the aPWD and lPWD $1\pi$- and $2\pi$-exchange ${\Lambda \textrm{NN}}$ matrix elements,

$\langle p^{\prime }_{12}, q^{\prime }_{3},\alpha ^{\prime }_{\Lambda \textrm{NN}} | V^{\Lambda \textrm{NN}} | p_{12}, q_3, \alpha _{\Lambda \textrm{NN}} \rangle$ in the partial-wave state $(1/2^+,0)$, as a function of the momentum $p_{12}$ for $p^{\prime }_{12}=q^{\prime }_{3}=q_3 =0.20550664$ $\hbox {fm }^{-1}$. Note that the matrix elements in Fig. 2 have been regularized employing a non-local regularization function of the form $f_{\Lambda }(p_{12},q_3) =\exp (-(p^2_{12} + \frac{3}{4} q_3^2)^2/\Lambda ^4)$ with a cutoff of $\Lambda =550$ MeV. Such a non-local regularization function has the advantage that it does not depend on the angles and therefore can be applied to the potential independently of the partial-wave decomposition. The so-called semi-local momentum-space (SMS) regularization developed by the Bochum group has however shown some advantages over the non-local regularization for the case of NN and 3NF forces [47]. The application of the SMS regularization to chiral YNN forces will be studied in [48]. Finally, Fig. 3 displays the $2\pi$-, $1\pi$-exchange and contact ${\Lambda \textrm{NN}}$ matrix elements, $\langle p^{\prime }_{12} q^{\prime }_{3} \, \alpha ^{\prime }_{{\Lambda \textrm{NN}}}=1 | V^{{\Lambda \textrm{NN}}} | p_{12} q_{3} \,\alpha _{\Lambda \textrm{NN}}=1\rangle$, in several partial-wave states $(J^{\pi }, T) = (1/2^+,0), (1/2^+,1), (3/2^+,0)$ and $(3/2^+,1)$ as a function of the hyperspherical coordinate $\xi ^2 = p^2_{12} + \frac{3}{4}q_3^2$ and at a hyperangle $\tan \theta = \frac{2}{\sqrt{3}}\, p_{12}/q_3 = \frac{\pi }{4}$. Also here the non-local regularization function with a cutoff of $\Lambda =550$ MeV has been applied to all potentials. The $V^{{\Lambda \textrm{NN}}}$ matrix elements in the $(3/2^+,1)$ state have been scaled by a factor of 10 in order to make them visible on the plot. In general, the matrix elements in the higher partial-wave states that are not shown in Fig. 3 are of at least two order of magnitude smaller than the ones in the $(1/2^+,0)$ state.

## $A=3-5$ hypernuclei with chiral ${\Lambda }$NN three-body forces

In this section, we will investigate the possible contributions of the chiral ${\Lambda }$NN potentials to the separation energies of $A=3-5$ hypernuclei. As one can see from Eqs. (4–6), the ${\Lambda }$NN potential is characterized by five LECs ($C'_1-C'_3$, $D'_1$, $D'_2$) which are difficult to determine due to the scarcity of the experimental data. However, using the decuplet saturation approximation the LECs can be qualitatively estimated. Specifically, they can be expressed in terms of contact interactions for $BB\rightarrow BB^*$, with pertinent LECs denoted by $H_i$ in Ref. [49]. Then we are left with only one unknown LEC ($H'=H_1+3H_2$) for the case of $V_{\Lambda \textrm{NN}}$ (and two LECs when both ${\Lambda }$NN and $\mathrm {\Sigma }$NN are considered) [49],

12 $$\begin{aligned}&C_{1}^{\prime } = C_{3}^{\prime } = \frac{H^{\prime 2}}{72 \Delta }, \qquad&C^{\prime }_2 =0, \\&D^{\prime }_{1} =0,&D_2^{\prime } = \frac{2C H^{\prime }}{9 \Delta },\\&3b_0 + b_D =0, \qquad&2b_2 + 3b_4 =-\frac{C^2}{\Delta }. \end{aligned}$$

Here $\Delta$ is the decuplet-octet baryon mass difference and $C = 3/4\, g_A \approx 0.95$ is the $B^*B\phi$ coupling constant [49]. As evidenced by Eq. (12), decuplet saturation fixes also the sub-leading meson-baryon LECs, i.e. the $b_i$. Note, however, that within decuplet saturation some LECs are zero and thus the most general structure of the YNN forces is not explored.

**Table 4:** Separation energies for s-shell ${\Lambda }$ hypernuclei without ${\Lambda }$NN 3BF and with $2\pi$-exchange, $1\pi$-exchange, or contact 3BF, respectively. All calculations are based on the SMS N^4^LO^+^(550) and NLO19(550) potentials for NN and YN, respectively, and on chiral ${\Lambda }$NN 3BFs with non-local regulator of $\Lambda =550$ MeV. For the NCSM calculations all potentials have been SRG-evolved at a flow parameter of $\lambda =1.88$ fm^–1^. Also, an uncertainty estimate for the results is provided

		w/o ${\Lambda }$NN	w. $2\pi$-ex ${\Lambda }$NN	w. $1\pi$-ex ${\Lambda }$NN	w. ct ${\Lambda }$NN	Exp. [52]
NCSM	$^3_{{\Lambda }}\textrm{H}$	$0.080 \pm 0.006$	$0.153 \pm 0.004$	$0.121 \pm 0.005$	$0.076 \pm 0.007$	$0.164 \pm 0.043$
FY		0.087	0.152	0.129	0.080	
NCSM	$^4_{{\Lambda }}\textrm{He}(0^+)$	$1.432 \pm 0.010$	$1.810 \pm 0.006$	$1.619 \pm 0.007$	$1.400 \pm 0.010$	$2.347 \pm 0.036$
	$^4_{{\Lambda }}\textrm{He}(1^+)$	$1.164 \pm 0.014$	$1.744 \pm 0.007$	$1.427 \pm 0.009$	$1.117 \pm 0.016$	$0.942 \pm 0.036$
	$^5_{{\Lambda }}\textrm{He}$	$3.174 \pm 0.020$	$4.618 \pm 0.011$	$3.757 \pm 0.034$	$2.961 \pm 0.031$	$3.102 \pm 0.030$

In principle, the LEC $H^\prime$ is to be determined via a fit to the binding energies of the s-shell hypernuclei, which is beyond the scope of this study. This issue will be thoroughly dealt with in a future investigation [48]. For our present purpose of exploring the chiral ${\Lambda }$NN 3BF, it is sufficient to assume a realistic scale for $H^{\prime }$. Therefore, we will adopt $H^{\prime } = 1/f^{2}_{0}$, as suggested in [49] based on dimensional scaling arguments, for all the calculations reported in this section. The separation energies for $A=3-5$ hypernuclei, computed using the two-body YN potential NLO19 with a cutoff of $\Lambda =550$ MeV in combination with the $2\pi$-, $1\pi$-exchange, or contact ${\Lambda }$NN potentials, are listed in Table 4. The semi-local momentum-space (SMS) NN potential at $\mathrm {N^4LO^+}$, likewise regularized with a cutoff of $\Lambda =550$ MeV, has been employed for describing the nuclear interaction. For $A=4$ ,5 hypernuclei also 3N forces contribute, for which we take the leading ($\mathrm {N^2LO}$) SMS regularized chiral 3NFs as specified for example in Table 1 of Ref. [40]. For the calculations with the NCSM, all interactions have been evolved with the similarity renormalization group (SRG) at a flow parameter of $\lambda =1.88$ fm^–1^ and the corresponding induced 3BFs (in 3N, ${\Lambda }$NN and $\mathrm {\Sigma }$NN) are included. As has been discussed, e.g., in [50, 51], the SRG-induced YNN forces can be much larger than what is expected for chiral YNN forces. Its size can be estimated by the SRG flow parameter dependence of the energy when the induced YNN forces are omitted. For $A=3$, 4 and 5, this has been found to be of the order of 300 keV, 1 MeV and 3.5 MeV, respectively [33]. However, we have carefully checked that, for the above flow parameter and using interactions up to the three-body level, the uncertainty due to omitted induced many-body forces is negligible (see also [40]). At the same time, NCSM calculations converge in reasonably sized model spaces [4, 39, 40]. As discussed in the previous section, the chiral ${\Lambda }$NN potential matrix elements at partial-wave states with the total angular momentum $J \ge 5/2$ are very small, their contributions to the binding energies are therefore expected to be insignificant. Indeed, we have observed that the ${\Lambda }$NN 3BFs with $J =5/2$ contribute only a few keV to the binding energies in the $A=4,5$ systems. Therefore, for the calculations for $A \ge 4$ systems, the ${\Lambda }$NN matrix elements $V^{{\Lambda \textrm{NN}}}$ with $J \ge 7/2$ will be omitted, whereas all the possible isospin states $T =0,1,2$ and parities are taken into account.

As already mentioned, for $\hbox {the }^3_{{\Lambda }}\textrm{H}$ system, we provide results from both the NCSM [39, 40] and the Faddeev approach [24]. The energies for the $A=4,5$ systems have been computed only within the NCSM. Clearly, the difference between the two $A=3$ results are smaller than the estimated uncertainty for the NCSM approach. The contribution of the contact potential

$V^{{\Lambda \textrm{NN}}}_{ct}$ to $B_{{\Lambda }}(^3_{{\Lambda }}\textrm{H})$ is negligibly small and repulsive,

whereas the $V^{{\Lambda \textrm{NN}}}_{2\pi }$ and $V^{{\Lambda \textrm{NN}}}_{1\pi }$ contributions are sizable and attractive, amounting to about 70 and 40 keV, respectively. Similarly, the effect of $V^{{\Lambda \textrm{NN}}}_{ct}$ to the binding energy $B_{{\Lambda }}(^4_{{\Lambda }}\textrm{He}, 0^+)$ is repulsive but with 30 keV rather insignificant. It becomes, however, moderately repulsive in $\hbox {the }^4_{{\Lambda }}\textrm{He}(1^+)$ $\hbox {and }^5_{{\Lambda }}\textrm{He}$ states, contributing about 50 and 200 keV, respectively. Interestingly, both the $1^+$ state in the $A=4$ system $\hbox {and }^5_{{\Lambda }}\textrm{He}$ are largely overbound with the $2\pi$- and $1\pi$-exchange ${\Lambda }$NN potentials, with respect to the present experimental information [52], while the ground state in $A=4$ remains underbound.

Since the sign of the LECs $C'_1$ and $C'_3$ parameterizing the contact interaction is completely fixed when decuplet saturation is assumed, cf. Eq. (12), it can be expected that the contribution from the contact terms remains repulsive for any combination of the LECs $H_1$ and $H_2$. Note however that choices that lead to a negative $H'$ are possible, which allow for a partial cancellation of $V^{{\Lambda \textrm{NN}}}_{2\pi }$ and $V^{{\Lambda \textrm{NN}}}_{1\pi }$. Anyway, a careful analysis of the $H'$ (or $H_1$ and $H_2$) dependence of the separation energies of the *s*-shell hypernuclei is beyond the scope of this work. Nonetheless, let us mention that exploratory calculations have shown that the inclusion of the chiral ${\Lambda }$NN-$\mathrm {\Sigma }$NN and $\mathrm {\Sigma }$NN three-body potentials alone does not lead to a qualitative change of the results for the light hypernuclei considered above. Rather, one has to really relax the constraints from decuplet saturation in order to reproduce the separation energies of $A=3-5$ hypernuclei, see [34] and [48].

## Conclusions

In this work, we examined two different approaches, lPWD and aPWD, to efficiently perform the partial-wave decomposition of three-body forces, for the chiral ${\Lambda }$NN (YNN) interactions. The ${\Lambda }$NN matrix elements of the two methods were compared with each other in detail. In general, an agreement of better than $0.1\%$ is observed for the $2\pi$-exchange potential, whereas the difference in all the $1\pi$-exchange and contact ${\Lambda }$NN potentials matrix elements is smaller than $0.5\%$. Such a benchmark provides a solid confirmation of the correctness of our implementations and is of importance for any future calculations that include the chiral YNN 3BFs.

As first application, we explored the possible impact of the leading chiral ${\Lambda }$NN potential on the separation energies of light hypernuclei. The sub-leading meson-baryon LECs appearing in the 2$\pi$-exchange 3BF and the LECs in the 1$\pi$-exchange contribution and the six-baryon contact term were estimated via decuplet saturation and assuming values for the LECs based on dimensional scaling arguments. It turned out that the weakly repulsive ${\Lambda }$NN contact interaction leads to a small contribution to the binding energies in all $A=3-5$ hypernuclei, whereas the two other contributions, $V^{{\Lambda \textrm{NN}}}_{2\pi }$ and $V^{\Lambda \textrm{NN}}_{1\pi }$, are moderately attractive for our choice of the only remaining LEC $H'$. The size of the individual contributions are significant even $\hbox {for }^3_\Lambda$H. This is somewhat surprising since estimates for chiral N$^2$LO contributions so far indicated negligible $\Lambda$NN force contributions [38, 40]. But the case studied here also leads to overbinding for the $J^\pi =1^+$ state $\hbox {of }^4_{{\Lambda }}\textrm{He}$ $\hbox {and }^5_{\Lambda }\textrm{He}$ $\hbox {while }^4_{\Lambda }\textrm{He}(0^+)$ is still clearly underbound. The interesting question whether one can determine an optimal combination of the LECs within the decuplet approximation so that all light hypernuclei are well described, should be and will be addressed in a future study. In such a study, it should also be addressed whether the $\Lambda$NN force contribution $\hbox {to }^3_\Lambda$H remains sizable.

## Data Availability

Data will be made available on reasonable request. [Author’s comment: The datasets generated during and/or analysed during the current study are available from the corresponding author on reasonable request.]

## Footnotes

1 The contribution from those permutations that yield identical results to the diagram in (c) is already included in Eq. (3), which explains for the factor of 18 instead of 36.

## Code availability Statement

Code/software will be made available on reasonable request. [Author’s comment: The code/software generated during and/or analysed during the current study is available from the corresponding author on reasonable request.]
